# (−)-Epigallocatechin-3-Gallate Inhibits the Chaperone Activity of *Plasmodium falciparum* Hsp70 Chaperones and Abrogates Their Association with Functional Partners

**DOI:** 10.3390/molecules22122139

**Published:** 2017-12-05

**Authors:** Tawanda Zininga, Lebogang Ramatsui, Pertunia Bveledzani Makhado, Stanley Makumire, Ikechukwu Achilinou, Heinrich Hoppe, Heini Dirr, Addmore Shonhai

**Affiliations:** 1 Department of Biochemistry, School of Mathematics and Natural Science, University of Venda, Thohoyandou 0950, South Africa; tzininga@gmail.com (T.Z.); lebogangramatsui@gmail.com (L.R.); bveledzanimakhado@gmail.com (P.B.M.); stanmakster@gmail.com (S.M.); 2 Protein Structure-Function Research Unit, School of Molecular & Cell Biology, University of the Witwatersrand, Johannesburg 2050, South Africa; Ikechukwu.Achilonu@wits.ac.za (I.A.); heinrich.dirr@wits.ac.za (H.D.); 3 Department of Biochemistry, Microbiology & Biotechnology, Rhodes University, Grahamstown 6140, South Africa; h.hoppe@ru.ac.za

**Keywords:** malaria, *Plasmodium falciparum*, (−)-epigallocatechin-3-gallate, PfHsp70-z, PfHsp70-1, molecular chaperone, functional associates, inhibitor

## Abstract

Heat shock proteins (Hsps), amongst them, Hsp70 and Hsp90 families, serve mainly as facilitators of protein folding (molecular chaperones) of the cell. The Hsp70 family of proteins represents one of the most important molecular chaperones in the cell. *Plasmodium falciparum*, the main agent of malaria, expresses six Hsp70 isoforms. Two (PfHsp70-1 and PfHsp70-z) of these localize to the parasite cytosol. PHsp70-1 is known to occur in a functional complex with another chaperone, PfHsp90 via a co-chaperone, *P. falciparum* Hsp70-Hsp90 organising protein (PfHop). (−)-Epigallocatechin-3-gallate (EGCG) is a green tea constituent that is thought to possess antiplasmodial activity. However, the mechanism by which EGCG exhibits antiplasmodial activity is not fully understood. A previous study proposed that EGCG binds to the N-terminal ATPase domain of Hsp70. In the current study, we overexpressed and purified recombinant forms of two *P. falciparum* cytosol localized Hsp70s (PfHsp70-1 and PfHsp70-z), and PfHop, a co-chaperone of PfHsp70-1. Using the surface plasmon resonance approach, we demonstrated that EGCG directly binds to the two Hsp70s. We further observed that binding of EGCG to the two proteins resulted in secondary and tertiary conformational changes. In addition, EGCG inhibited the ATPase and chaperone function of the two proteins. Furthermore, EGCG abrogated association of the two Hsp70s with their functional partners. Using parasites cultured in vitro at the blood stages, we observed that 2.9 µM EGCG suppressed 50% *P. falciparum* parasite growth (IC_50_). Our findings demonstrate that EGCG directly binds to PfHsp70-1 and PfHsp70-z to inhibit both the ATPase and chaperone functions of the proteins. Our study constitutes the first direct evidence suggesting that the antiplasmodial activity of EGCG is at least in part accounted for by its inhibition of Hsp70 function.

## 1. Introduction

Malaria remains the world’s most devastating disease for most people living in tropical areas, accounting for 429,000 deaths across the globe [1]. Unfortunately, parasite resistance to antimalarial drugs is steadily on the rise. For this reason, new antimalarial treatment options are urgently needed towards control and elimination of the disease. Heat shock proteins (Hsps) are generally regarded as ‘molecular chaperones’ because of their role in facilitating the folding of other proteins. Since cellular stress impacts adversely on proteostasis, select Hsps are upregulated during cellular stress in order to reverse and prevent protein misfolding. The main agent of malaria, *Plasmodium falciparum* expresses several classes of Hsps. Given their role in maintaining proteostasis, it is not surprising that some Hsps from *P. falciparum* are essential [2]. In addition, some of them are implicated in parasite drug resistance and the expression levels of some of them is thought to regulate clinical malaria progression [3]. Despite their conservation, it has been proposed that it is possible to selectively target Hsps of parasitic origin with minimum effects on their human counterparts [4,5].

The Hsp70 family represents one of the major families of molecular chaperones. Hsp70 consists of an N-terminal nucleotide binding domain (NBD) that is highly conserved. The NBD/ATPase domain is connected to the C-terminal peptide binding domain (PBD) via a highly-conserved linker segment which facilitates allosteric communication between the two domains [6]. In the ATP bound state, the PBD of Hsp70 assumes an ‘open’ conformation which allows the substrate to be released [7]. On the other hand, the ADP bound state of Hsp70 possesses a high affinity for the substrate. For this reason, the hydrolysis of ATP to ADP is important towards facilitating holding of the substrate by Hsp70. However, Hsp70 possesses weak basal ATPase activity [8]. Hsp40 represents a distinct group of molecules whose main role is to serve as substrate scanners of Hsp70 [9]. Hsp40s are also known to upregulate the ATPase activity of Hsp70 [9]. In order for the functional cycle of Hsp70 to continue, facilitation of nucleotide exchange (ADP release to allow ATP binding) is required. In eukaryotic cells, Hsp70 nucleotide exchange is facilitated by various protein families, amongst them, the Hsp110 family [10]. Hsp110 chaperones are Hsp70-like proteins. However, they are distinct in that they are larger than canonical Hsp70s. Their large size is attributed to their possession of an extended lid segment that covers the peptide binding cavity and which occurs at the C-terminus of the protein [11]. In addition, canonical Hsp70s are known to not only prevent protein aggregation, but they also refold misfolded proteins [12]. On the other hand, Hsp110 chaperones are capable of binding onto misfolding proteins to prevent aggregation, but they are incapable of refolding proteins [13].

*P. falciparum* expresses six Hsp70 like proteins. Two (PfHsp70-1; PF3D7_0818900 and PfHsp70-z; PF3D7_0708800) of which occur in the cytosol [14,15,16]. PfHsp70-1 is thought to belong to the canonical group of Hsp70s, whereas PfHsp70-z belongs to the Hsp110 family [16]. Inhibition of PfHsp70 by select small molecules has been linked to parasite death [17], suggesting that the protein is essential. A gene knock-out study showed that PfHsp70-z is essential for parasite growth at the blood stage [18]. Expression of the two proteins at the clinical phase of malaria has been linked to disease prognosis. Apart from being essential, cooperation of PfHsp70-1 with PfHsp70-z is thought to be important for parasite survival [16]. To this end, our previous work demonstrated the existence of PfHsp70-1 and PfHsp70-z in nucleotide-dependent complexes [16]. It is thought that PfHsp70-z may associate with PfHsp70-1 to facilitate the nucleotide exchange function of the latter [16]. In addition, we previously established that whereas the chaperone activity (suppression of protein aggregation) of PfHsp70-1 is nucleotide-dependent, the activity of PfHsp70-z is nucleotide-independent [16]. Altogether, our previous findings suggest that the two chaperones exhibit distinct functional features through which they play their cytoprotective functions. It has been proposed that PfHsp70-z has propensity to bind asparagine-rich repeat proteins which constitute about a quarter of the parasite proteome. Given the fact that asparagine-rich repeat proteins are generally aggregation prone, it appears that both PfHsp70-1 and PfHsp70-z may serve as a buffer against the stress that characterizes the development of the parasite under the physiologically-divergent development phases that it undergoes in the human host.

(−)-Epigallocatechin-3-gallate (EGCG) is a major catechin found in green tea. EGCG has been proposed as a potential drug for several diseases, including cancer [19]. It has been proposed that EGCG competitively inhibits ATP binding on Hsp70 [20]. EGCG has been reported to exhibit antiplasmodial activity and is known to augment the antimalarial function of artemisinin [21,22].

Heat shock proteins occur in networks with their functional chaperone partners and regulatory partners (co-chaperones). In addition, Hsp70 chaperones are allosteric molecules. Thus, modulation of Hsp70s by small molecule inhibitors impacts on their global conformation, and this influences their association with functional partners [23]. For example, we recently observed that polymyxin B binds to PfHsp70-1 to inhibit its chaperone function [23]. In the same study, we observed that polymyxin B abrogates association of PfHsp70-1 with PfHsp70-z and a co-chaperone, *P. falciparum* Hsp70-Hsp90 organizing protein (PfHop). Although EGCG is proposed to possess antiplasmodial activity, its mechanism of action against malaria parasites has not been explored. Previous studies suggested that EGCG may target an enoyl-acyl carrier protein reductase of *P. falciparum* (PfENR) [24]. In the current study, we proposed that EGCG may potentially target Hsp70 function to effect antiplasmodial action. It has been observed that most compounds that possess antimalarial activity target the oxidation-reduction pathway [25,26] which is closely linked to the cell’s stress response. Since Hsp70 is important for parasite cytoprotection, particularly against cell stress, and is implicated in the development of malaria pathogenicity, identifying molecules that target this chaperone is important. In the current study, we investigated the effect of EGCG on the structure-function features of cytosolic *P. falciparum* Hsp70s (PfHsp70-1 and PfHsp70-z). We observed that that EGCG is capable of binding to both PfHsp70-1 and PfHsp70-z to inhibit both their chaperone function and ATPase activity, respectively. Furthermore, EGCG binding perturbed the conformation of PfHsp70-1, leading to abrogation of its association with both PfHsp70-z and PfHop. Our findings suggest that one mechanism in which EGCG manifests its antiplasmodial action is through targeting Hsp70 function.

## 2. Results

### 2.1. EGCG Directly Binds to Both PfHsp70-1 and PfHsp70-z

**Figure 1:**
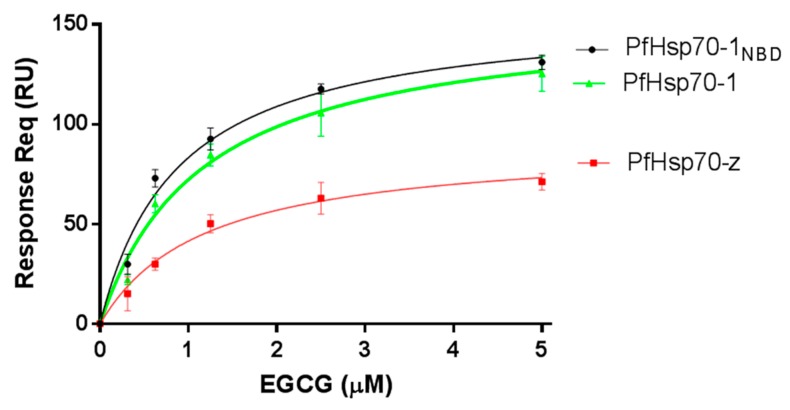
EGCG is capable of binding both PfHsp70-1 and PfHsp70-z. The ligands (PfHsp70-1/PfHsp70-1_NBD_) were immobilised onto the chip twice at a concentration of 0.5 µg/mL and 1 µg/mL, respectively, per run. The interaction between ligand and analyte (PfHsp70-z) was determined at equilibrium using SPR analysis. The assay was conducted in the presence of variable levels of EGCG. The data shown represent three independently conducted assays run in duplicates.

Surface plasmon resonance (SPR)-based analysis was used to assess if EGCG has the capability to bind to the Hsp70 chaperones. Firstly, we validated the functional specificity of the Hsp70 chaperones to bind nucleotides (ATP/ADP) (Figure 1, Table 1), as has been previously reported [16]. The SPR data reflected that EGCG binds to the two proteins in a concentration dependent fashion (Figure 1).

The equilibrium binding affinities (Table 1) show that EGCG binds to either PfHsp70-1 or its subdomain (PfHsp70-1_NBD_) in the lower micromolar range. Furthermore, its affinity for either PfHsp70-1 or its subdomain (PfHsp70-1_NBD_) was within the same order of magnitude (Table 1). Since EGCG binds to full-length PfHsp70-1 and its truncated form (PfHsp70-1_NBD_) with comparable affinity, this demonstrates that, in the absence of bound substrate, the C-terminal PBD of PfHsp70-1 has little influence on the steady-state EGCG binding kinetics. This suggests that the NBD is the minimal domain that is required for EGCG binding. This is consistent with a previous report [20]. Furthermore, the binding kinetics data suggest that although PfHsp70-1 and PfHsp70-z both exhibit high affinity (in the micromolar range) for EGCG (Figure 1, Table 1), PfHsp70-1 binds EGCG with much higher affinity. Furthermore, the data suggest that EGCG binds to either PfHsp70-1 or PfHsp70-z with approximately 10-fold higher affinity than ATP. This is in agreement with an independent study which previously proposed that EGCG exhibits higher affinity than ATP for Hsp70 [20].

### 2.2. EGCG Induces Conformational Changes on Cytosolic P. falciparum Hsp70s

**Figure 2:**
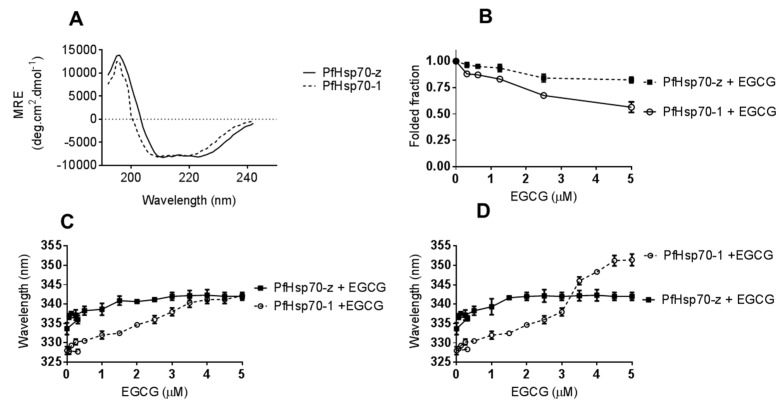
EGCG interacts with Hsp70 to induce conformational changes. The general folds of PfHsp70-1 and PfHsp70-z in the absence of EGCG was monitored by CD spectrometry (**A**). The folded fractions of PfHsp70-1 and PfHsp70-z in the presence of varying levels of EGCG were determined by CD spectrometry (**B**). Subsequently, 2 µM PfHsp70-1/PfHsp70-z was exposed to variable levels of EGCG and the resultant respective red shifts for (**C**) tryptophan fluorescence, and (**D**) combined tryptophan-tyrosine fluorescence, respectively, are shown.

Analyses of the secondary and tertiary structures of the recombinant forms of cytosolic *P. falciparum* Hsp70s were conducted using CD and fluorescence spectroscopy, respectively (Figure 2). The general folds of the two proteins show a similar orientation with minor variations on the compositions (Figure 2A). In the absence of nucleotides, both PfHsp70-1 and PfHsp70-z exhibited secondary structural profiles (Figure 2A) similar to previously reported findings [27]). Effects of EGCG on the secondary structural fold of the parasite proteins were investigated by exposing them to various levels of EGCG and monitoring the changes in the folded fraction at 222 nm (Figure 2B). At the highest concentration of EGCG used (5 μM), the folded fraction was 65%. In comparison to PfHsp70-1, PfHsp70-z seems more resilient to structural perturbation by EGCG (Figure 2B). Tertiary conformational changes were monitored by tryptophan fluorescence as both proteins possess tryptophan residues. In order to avoid photo-bleaching the preparations were only measured once and several preparations scanned. Upon monitoring the peak wavelength for both tryptophan and tyrosine fluorescence a red shift was observed (Figure 2C,D). Thus, EGCG causes structural changes to PfHsp70-1 and PfHsp70-z in a similar fashion to that induced by the cyclic peptide, polymyxin B, as we previously reported [27].

### 2.3. EGCG Inhibits the ATPase Activities of PfHsp70-1 and PfHsp70-z

**Figure 3:**
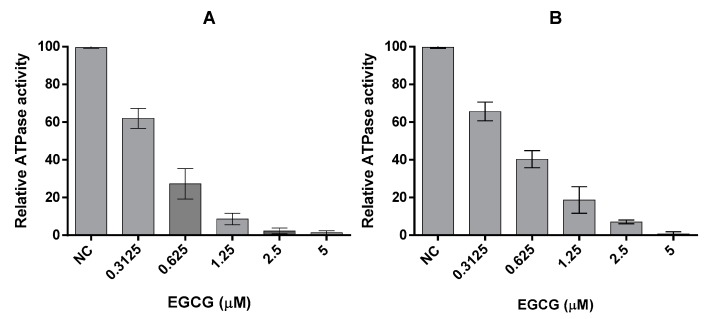
EGCG inhibits the intrinsic ATPase activity of Hsp70. The effect of EGCG on the basal ATPase activities of (**A**) PfHsp70-1 and (**B**) PfHsp70-z were investigated. The released P*i* was monitored at 595 nm using a direct colourimetric assay. The experiment was carried out under ATP saturation (5 mM) in the presence of 0.4 µM PfHsp70-1/PfHsp70-z.

We previously reported that PfHsp70-1 possesses slightly higher affinity for ATP than PfHsp70-z and their respective basal ATPase are within the same range of magnitude [16]. In the current study, the basal ATPase for the two cytosolic *P. falciparum* Hsp70s were determined using saturating levels of ATP concentration (5 mM) (Figure 3, Table 2). The assay was repeated in the presence of variable amounts of EGCG up to 5 μM (Figure 3). The Michaelis-Menten curves determined show loss of activity in response to increasing levels of EGCG (Table 2). While the *V_max_* was constant, *K_m_* increased in in the presence of higher levels of EGCG (Table 2), suggesting that EGCG may have competitively inhibited the two proteins with Ki of 1.72 (±0.62) μM for PfHsp70-1 and 2.34 (±0.84) μM for PfHsp70-z, respectively. Furthermore, the fact that the N-terminal NBD constitutes the minimum structural entity of PfHsp70-1 required to bind EGCG suggests that EGCG may compete with ATP for binding to this subdomain, as previously proposed [20].

### 2.4. EGCG Suppresses the Chaperone Activities of both PfHsp70-1 and PfHsp70-z In Vitro

**Figure 4:**
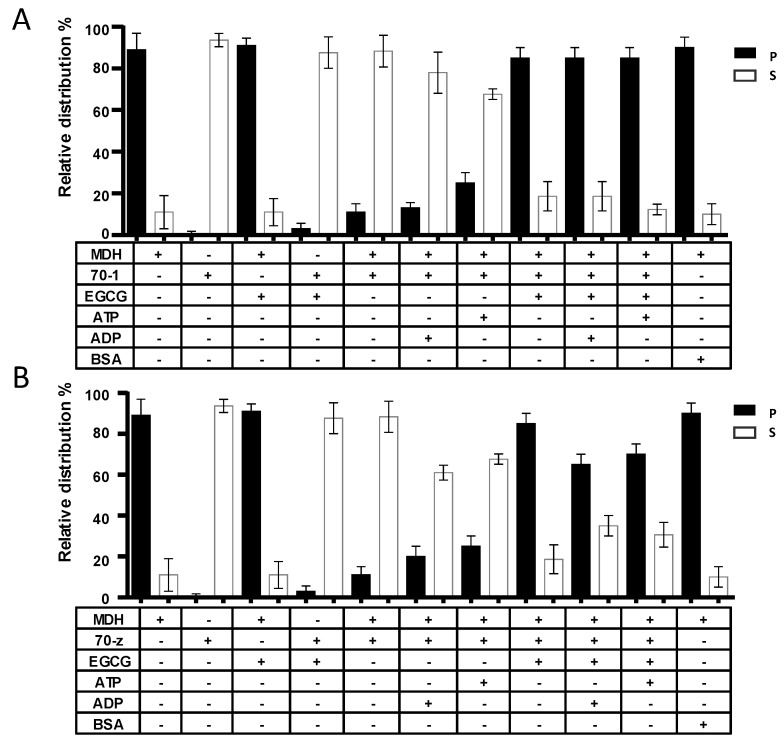
EGCG suppresses chaperone function of PfHsp70-1and PfHsp70-z. The independent chaperone functions of PfHsp70-1 (**A**) and PfHsp70-z (**B**) were investigated by monitoring the heat-induced aggregation of MDH in vitro at 48 °C, followed by quantitation of the pellet (P) and soluble (S) fractions, respectively. The assay was repeated in the presence of EGCG and/or nucleotides. The activity of PfHsp70-1 and PfHsp70-z in the presence of EGCG when compared to the activity of the respective protein either in nucleotide-free state or ADP bound state was statistically significant (*p* < 0.005). Statistical analysis was conducted using one way ANOVA. Standard deviations obtained from three replicate assays are shown.

**Figure 5:**
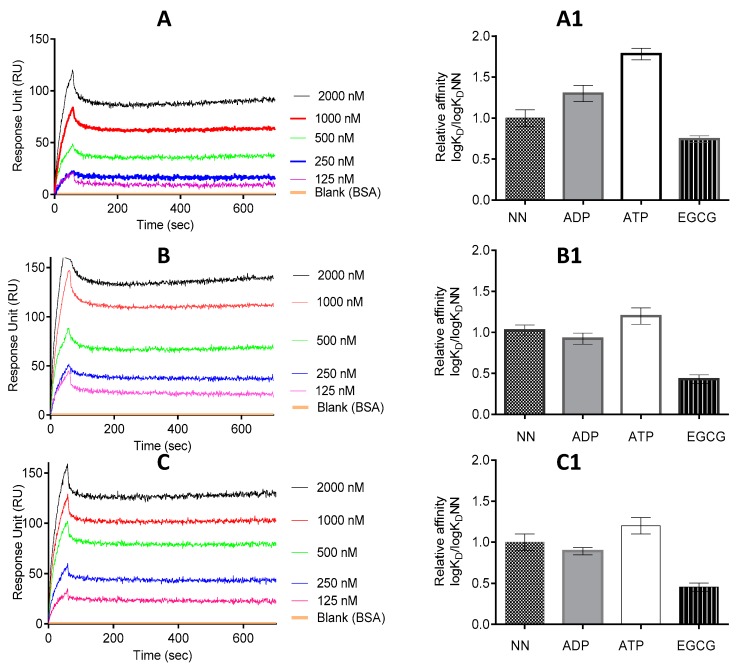
EGCG abrogates interaction of PfHsp70-1 with both PfHsp70-z and PfHop. Shown are the representative SPR sensorgrams representing the interaction of PfHsp70-1/PfHsp70-1_NBD_ and PfHsp70-z as investigated under varying levels of EGCG. The data represent global fitting obtained under conditions in which ligand and analyte were interchanged (**A**). The assay was repeated for PfHsp70-1/PfHsp70-1_NBD_ (as ligand) versus PfHsp70-z (as analyte) (**B**); and PfHsp70-1 (ligand) versus PfHop (analyte) (**C**). The assay was conducted under the following conditions: absence of nucleotides (NN); presence of either 5 mM ATP/ADP or 2.5 μM EGCG, respectively. The relative affinities for interaction of the following protein pairs are shown: PfHsp70-1-PfHsp70-z (**A1**); PfHsp70-1_NBD_-PfHsp70-z (**B1**); and PfHsp70-1-PfHop (**C1**). The relative affinities shown represent log*K_D_* normalized to log*K_D_*NN in each case.

In a previous study, we demonstrated that both PfHsp70-1 and PfHsp70-z are heat stable and that PfHsp70-z is more resilient to heat stress than PfHsp70-1 [16]. In addition, both proteins are capable of suppressing heat-induced aggregation of model proteins, such as malate dehydrogenase (MDH) [23,28]. In the current study, MDH was subjected to heat stress at 48 °C and, as expected, the protein aggregated in the absence of chaperones (Figure 4). In the presence of a non-chaperone protein (BSA), MDH also aggregated in response to heat stress. As expected, the introduction of either of the two chaperones resulted in the suppression of the heat-induced aggregation of MDH. As we previously observed [16], the introduction of ADP to the reaction in which either PfHsp70-1 or PfHsp70-z was present did not alter the chaperone activity of the respective protein (Figure 4). Again, in line with previous observations [16,28], the addition of ATP, while not affecting the activity of PfHsp70-z, did inhibit the activity of PfHsp70-1 (Figure 5). Next, we investigated the influence of EGCG on the chaperone function of PfHsp70-1 and PfHsp70-z. In the absence of the chaperones, MDH fully aggregated both in the absence and presence of 2.5 µM EGCG (Figure 4). However, both PfHsp70-1 and PfHsp70-z remained in solution in the presence of up to 5 µM EGCG. Having confirmed that the two proteins were stable in the presence of up to 5 µM EGCG, we subsequently repeated the assay in the presence of 2.5 µM EGCG and either of the two chaperones plus MDH. Interestingly, in the presence of EGCG the capability of both PfHsp70-1 and PfHsp70-z to suppress the heat-induced aggregation of MDH was abrogated. Indeed, the effect of EGCG on the chaperone activity of PfHsp70-1 resembled that of ATP [28], suggesting that both ATP and EGCG may act in the same manner to inhibit the protein. Although EGCG suppressed the chaperone activity of PfHsp70-z, the inclusion of ADP/ATP to the reaction mix slightly rescued the aggregation of MDH (Figure 4B). We observed a similar phenomenon in which either ADP/ATP slightly reduced the inhibition of the chaperone activity of PfHsp70-z by the peptide antibiotic, polymyxin B [23].

### 2.5. EGCG Inhibits the on and off Rates of PfHsp70-1 with Its Functional Associates

The effect of EGCG on the interaction of PfHsp70-1 with PfHsp70-z was investigated using SPR analysis. SPR analysis involves determination of the association rate constant, *Ka* (increase in the RU signal) and dissociation rate constant, *Kd* (decrease in the RU signal) obtained from the analysis of the association of interacting pairs of molecules. The *Kd* and *Ka* values are then used to determine the affinity constant *KD*, which is equal to *Kd*/*Ka*. The RU signals and *KD* values we obtained are summarized in Figure 5 and Table 3. Our previous studies demonstrated that PfHsp70-1 associates with PfHsp70-z either in the presence or absence of nucleotide [16,23]. However, compared to the scenario in which nucleotide was absent, or in which ADP was included, the inclusion of ATP was the most effective in promoting the association of PfHsp70-1 and PfHsp70-z (Figure 5, Table 3) as previously observed [16]. We then proceeded to investigate the effect of EGCG on the association of PfHsp70-1 and PfHsp70-z in the absence or presence of the respective nucleotide.

As expected, ATP promoted the interaction of PfHsp70-z with both full length PfHsp70-1 and PfHsp70-1_NBD_ (Figure 5, Table 3), as previously observed [16]. However, the addition of EGCG to the reaction mix resulted in the abrogation of the interaction between PfHsp70-z with PfHsp70-1/PfHsp70-1_NBD_, either in the absence or presence of nucleotide (Figure 5A1,B1).

PfHsp70-1 is known to associate with the co-chaperone, PfHop which functionally links it to another parasite chaperone, PfHsp90 [29,30]. Thus, we further investigated the effect of EGCG on this association. As we previously reported [30], the association of PfHsp70-1 and PfHop occurred in the absence as well as in the presence of ATP/ADP (Figure 5C, Table 3). Altogether, ADP is known to promote the association most effectively (Figure 5C1, Table 3, [30]). However, the relative affinities of the association of PfHsp70-1 and PfHop obtained either in the absence or presence of ATP/ADP were generally reduced upon introduction of EGCG (Figure 5C1).

The interaction kinetics represented by the equilibrium constant (*K_D_*) were determined by SPR analysis. The status of PfHsp70-z/PfHop and PfHsp70-1/PfHsp70-1_NBD_ as ligand and analyte, respectively, were alternated. The ligand was the respective immobilised protein on the chip surface and the analyte was the respective protein injected at a flow rate of 50 µL/min. Data were analysed by using readings obtained for the running buffer (in the presence of nucleotides, but lacking protein analyte) as the baseline. Data are represented as the mean plus/minus standard deviation. Chi square (*χ^2^*) values represent the Langmuir curve-fitting residuals. The statistical analysis was conducted using one-way ANOVA. * *p* < 0.005; ** *p <* 0.001 represent, the respective levels of significance for affinities observed in the presence of EGCG compared to the rest of the experimental conditions in each case.

### 2.6. EGCG Exhibits Antiplasmodial Activity

**Figure 6:**
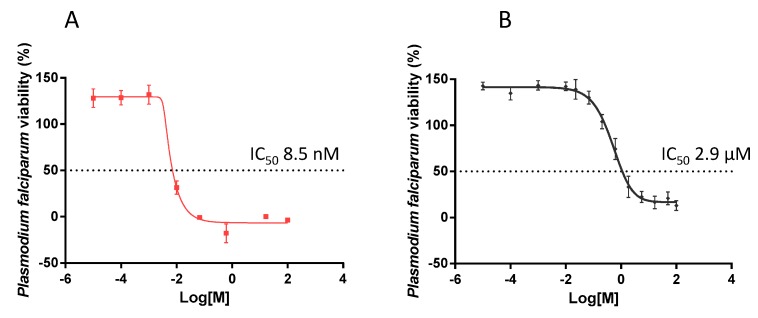
Normalized dose response curve for the in vitro susceptibility of *P. falciparum* to EGCG. The in vitro antiplasmodial activity of EGCG was investigated using a pLDH ELISA assay. The assay was conducted on *P. falciparum* 3D7 cells maintained at the blood stages in the presence of EGCG. Chloroquine was used as a control (IC_50_ of 8.5 nM). The curves represent data obtained from assays conducted in the presence of choloroquine (**A**) or EGCG (**B**), respectively. IC_50_ values were calculated from the generated dose response curves obtained using GraphPad Prism 6. The experiment was performed in duplicate wells. Standard deviations obtained from three replicate assays are shown.

The antiplasmodial activity of EGCG was investigated by monitoring the activity of lactate dehydrogenase (pLDH) of *P. falciparum* 3D7 cultured in vitro, as previously described [31]. It was important to validate parasite susceptibility to an established antimalarial compound. To this end we exposed the parasites to chloroquine which, at a final concentration of 8.5 nM, did inhibit parasite growth by 50% (IC_50_). The antiplasmodial activity of chloroquine that we observed (Figure 6A) is comparable to an independently-reported IC_50_ value [32]. The growth of parasites in the presence of EGCG was reduced in a concentration-dependent manner (Figure 6B). EGCG inhibited 50% of parasite growth (IC_50_) at a concentration of 2.9 µM. This finding further suggests that EGCG is a potent antiplasmodial agent, as previously suggested [21].

## 3. Discussion

EGCG has been reported to possess antimicrobial activity and offers promise towards addressing the growing threat of multidrug resistance among pathogens [20,33]. It has been proposed that EGCG binds to the NBD of Hsp70 [20]. This study, for the first time, demonstrates that EGCG binds directly to two *Plasmodium falciparum* cytosol-resident Hsp70s that are thought to be structurally and functionally distinct [14,16]. First, we established that EGCG binds primarily to the NBD of PfHsp70-1 (Figure 1). In addition, we further compared the affinity of EGCG for PfHsp70-1 and PfHsp70-z. We established that EGCG bound to PfHsp70-1 with higher affinity compared to its affinity for PfHsp70-z. This suggests that EGCG is capable of distinguishing between the two Hsp70s in spite of them possessing highly-conserved nucleotide binding domains (NBDs). Our findings further demonstrate that EGCG binds to the two proteins with higher affinity than ATP (Table 1). This suggests that EGCG binds to the NBD of Hsp70 to competitively exclude ATP.

We recently reported that the cyclic peptide, polymyxin B binds to both PfHsp70-1 and PfHsp70-z resulting in secondary and tertiary structural perturbations [23]. Similarly, in the current study, we observed that EGCG binding modulated the structural conformations of the two chaperones (Figure 2). The effect of EGCG was more enhanced on PfHsp70-1 than it was on PfHsp70-z which is in line with our previous report that PfHsp70-z is more resilient to heat stress and urea denaturation than PfHsp70-1 [16]. We further investigated if the structural changes would impact on the association of the two proteins with their functional partners [16]. We observed that EGCG abrogated PfHsp70-1:PfHsp70-z and PfHsp70-1:PfHop-based associations, both in the presence and absence of nucleotide (Figure 5, Table 3). We previously observed a similar phenomenon in which polymyxin B inhibition of PfHsp70-1 abrogated its interaction with PfHsp70-z and PfHop [23]. Therefore, our current findings, suggest that inhibitors of Hsp70s tend to structurally perturb the chaperones to abrogate their association with functional partners. The inhibition of the association between PfHsp70-1 and PfHsp70-z is important as the latter is thought to be the sole nucleotide exchange factor of PfHsp70-1 [16]. In addition, we previously reported the existence of PfHsp70-1 and PfHsp90 in a complex that is mediated by PfHop as a module [29]. As the current study has established that EGCG inhibits PfHsp70-1 directly and also abrogates its association with PfHop and PfHsp70-z, the data suggest that EGCG may be a versatile inhibitor of the molecular chaperone system of the cell.

We also investigated the effect of EGCG on the functions of the two proteins. In a previous study we reported that PfHsp70-1 possesses slightly higher affinity for ATP than PfHsp70-z and their respective basal ATPase activities are within the same range of magnitude [16]. In the current study, we demonstrated that EGCG binds to the two proteins to inhibit their basal ATPase activities. This study further established that EGCG inhibits the chaperone function of both PfHsp70-1 and PfHsp70-z in vitro (Figure 4). Whereas PfHsp70-z exhibits nucleotide-independent chaperone activity, the chaperone activity (suppression of model protein aggregation in vitro) of PfHsp70-1 is inhibited by ATP [16,28]. Our current data demonstrate that EGCG inhibits the chaperone activity of both PfHsp70-1 and PfHsp70-z. However, both nucleotides (ATP/ADP) were capable of slightly reversing the effect of EGCG on MDH aggregation in the presence of PfHsp70-z. On the other hand, the nucleotides did not appear to reverse the effect of EGCG on the heat-induced aggregation of MDH in the presence of PfHsp70-1 (Figure 4). Since our current study demonstrated that EGCG binds to PfHsp70-1 with higher affinity than it has for PfHsp70-z, this could explain why the EGCG exhibits a more enhanced effect on PfHsp70-1 than on PfHsp70-z.

We further demonstrated that EGCG inhibited growth of *P. falciparum* 3D7 parasites maintained at the red blood stage (Figure 6). EGCG inhibited 50% of parasite growth (IC_50_) at a concentration of 2.9 µM. This is in line with previous reports suggesting that EGCG exhibits antimalarial activity [22,24]. It has been suggested that EGCG may target an enoyl-acyl carrier protein reductase of *P. falciparum* (PfENR) to elicit its antimalarial activity [24]. Our current findings suggest that inhibition of Hsp70 by EGCG may partly account for its antiplasmodial action. In addition, we established that EGCG abrogates interaction of Hsp70 with its functional associates. It has been proposed that almost 25% of the *P. falciparum* proteome possess asparagine repeat-rich regions, a factor that increases the propensity of these proteins to aggregate under heat stress [18]. For this reason, the role of Hsp70 in stabilising the proteome of the parasite is crucial to its survival. The prominent role of Hsp70 in the development of the parasite makes this molecular chaperone a promising antiplasmodial drug target.

## 4. Materials and Methods

### 4.1. Materials

Unless, otherwise specified, chemical reagents used in this study were purchased from Merck Chemicals (Darmstadt, Germany), Melford (Suffolk, UK), and Sigma-Aldrich (St. Louis, MO, USA). EGCG was purchased from Sigma-Aldrich (St. Louis, MO, USA).

### 4.2. Expression and Purification of Recombinant Proteins

Constructs encoding PfHsp70-1 (pQE30/PfHsp70-1 [14]) and PfHsp70-z (pQE30/PfHsp70-z [27]) were used for the expression of recombinant PfHsp70-1 and PfHsp70-z proteins. The recombinant proteins were expressed in *Escherichia coli* XL1 Blue and JM109 cells, respectively, following previously described protocols [27,28]. A construct expressing PfHop (pQE30/PfHop) was used for the production of PfHop, as previously described [29]. In addition, we also expressed a truncated version of PfHsp70-1 constituting its ATPase/nucleotide binding domain (PfHsp70-1_NBD_), as previously described [29]. The recombinant proteins were expressed with N-terminally attached polyhistidine tags which facilitated purification using affinity chromatography, as previously described [16,27,29].

### 4.3. Determination of Binding Affinity of EGCG for PfHsp70-1 and PfHsp70-z

The binding affinity of EGCG for the two *P. falciparum* Hsp70s was determined using a Bio-Rad ProteOn XPR36 system (Hercules, CA, USA), as previously described [16]. Briefly, PfHsp70-1, PfHsp70-1_NBD_, and PfHsp70-z (as ligands) were separately immobilized onto an HTE chip at concentrations of 0.5 μg/mL and 1 μg/mL, respectively, in order to validate the data. As analyte controls, ATP/ADP [16] were prepared at final concentrations of 0, 125, 250, 500, 1000, and 2000 nM, respectively, and were injected at 100 μL/min into each horizontal channel. In order to determine the association of EGCG with the respective ligand, the EGCG stock solution was prepared in deionised water. Aliquots of EGCG were prepared at final concentrations of 0, 125, 250, 500, 1000, and 2000 nM and injected at 100 μL/min into each horizontal channel. Association was allowed for 2 min and dissociation was monitored for 8 min. Data collected was double referenced using a buffer blank (buffer without EGCG) and a channel in which protein was excluded. Steady-state equilibrium constant data were processed and analysed using BioRad ProteOn Manager Version 3.1.0.6 (Hercules, CA, USA) and GE Healthcare Biosciences BIA evaluation Version 4.1.1 software (Uppsala, Sweden).

### 4.4. Analysis of the Effect of EGCG on the Conformations of PfHsp70-1 and PfHsp70-z

To determine the effects of EGCG on the secondary structure of the recombinant proteins, the folding status of the respective protein was monitored using the Far–UV J-1500 CD spectrometer (JASCO Ltd., Dunmow, UK) set at 180–260 nm, as previously described [27]. Briefly, 2 μM of the respective recombinant protein (PfHsp70-1/PfHsp70-z) was suspended in 20 mM Tris-HCl pH 7.4, 100 mM sodium fluoride buffer and the reaction mix was placed in a 0.1 cm path-length quartz cuvette (Hellma, Müllheim, Germany). The assay was conducted in the presence of varying amounts (0–5 µM) of EGCG and the data were used to generate a curve representing the general folded fraction of the respective protein, as previously described [27].

We further exposed the two proteins to varying amounts (0–5 µM) of EGCG and monitored he perturbation of the tertiary structural conformations of the proteins using both tyrosine and tryptophan fluorescence, as previously described [27]. The respective recombinant protein (PfHsp70-1 of PfHsp70-z) at a final concentration of 0.45 μg/mL was incubated in assay buffer A (25 mM HEPES-KOH pH 7.5, 100 mM KCl, 10 mM MgOAc) at 20 °C either in the absence or presence of variable EGCG levels (0–5 μM). The assay was repeated in the presence of either 5 mM ATP or 5 mM ADP were used in place of EGCG as they represent known modulators of Hsp70 structure [16,27]. Tryptophan fluorescence spectra were recorded between 300 nm and 400 nm after initial excitation at 295 nm using a JASCO FP-6300 spectrofluorometer (Dunmow, UK). The combined fluorescence of tyrosine and tryptophan was monitored after initial excitation at 285 nm. Subsequently, fluorescence was monitored at wavelengths between 300 nm and 400 nm using a JASCO FP-6300 spectrofluorometer. The resultant spectra were averaged for least 15 scans and processed after subtraction of the baseline scan (buffer in which protein was excluded). A control consisting of a buffer containing EGCG, but lacking protein was used to account for baseline fluorescence.

### 4.5. Investigation of the Effect of EGCG on the ATPase Function of PfHsp70-1 and PfHsp70-z

The basal ATPase activities of the parasite Hsp70s was assessed by a colourimetric-based method, as previously described [27]. Briefly, 0.4 µM of recombinant proteins (PfHsp70-1/PfHsp70-z) was incubated in buffer HKMD (10 mM HEPES-KOH pH 7.5, 100 mM KCl, 2 mM MgCl2, 0.5 mM DTT) at 37 °C. The reaction was started by the addition of saturating 5 mM ATP and samples were collected after every 30 min for 4 h. The released inorganic phosphate was detected by adding 1.25% ammonium molybdate and 9% ascorbic acid and monitored using a M3 SpectraMax (Molecular Devices, Sunnyvale, CA, USA) spectrophotometer at 660 nm. To determine the effect of EGCG on the ATPase activity of parasite Hsp70s, the assay was repeated in the presence of varying amounts (0–5 µM) of EGCG. As controls, boiled Hsp70 protein was used to account for spontaneous ATP hydrolysis, and BSA was used as a non-chaperone control.

### 4.6. Investigation of the Effect of EGCG on the Chaperone Function of PfHsp70-1 and PfHsp70-z

The chaperone function of parasite Hsp70s was investigated by monitoring heat-induced aggregation of a model protein, malate dehydrogenase (MDH) from porcine heart (Sigma-Aldrich), as previously described [16], with minor modifications. Before conducting the assays, we first established that both purified preparations of PfHsp70-1 and PfHsp70-z were heat-stable at 48 °C. After this, we then investigated the effects of the chaperones (PfHsp70-1/PfHsp70-z) on the heat-induced aggregation of MDH. To determine the effects of EGCG on the chaperone function of the two proteins, the assay was repeated in the presence of 2.5 µM of EGCG. EGCG was used at this concentration (2.5 µM) because this was the lowest concentration at which it inhibited the function of the chaperones. Furthermore, within this concentration range EGCG did not promote the heat-induced aggregation of MDH. We previously showed that ATP inhibits the capacity of PfHsp70-1 to suppress heat-induced aggregation of MDH and, on the other hand, the chaperone activity of PfHsp70-z is nucleotide-independent [16]. For this reason, the assay was repeated in the absence of both EGCG and nucleotide (0 µM EGCG + 0 mM ADP/ATP), or the presence of nucleotide (0 µM EGCG + 5 mM ADP/ATP), as well as in the presence of EGCG (2.5 µM EGCG + 0 mM ADP/ATP). For statistical analysis, a one-way ANOVA test was conducted using GraphPad Prism 6 (San Diego, CA, USA).

### 4.7. Analysis of the Effect of EGCG on Association of PfHsp70-1 with Either PfHsp70-z or PfHop

The interaction between PfHsp70-1 with its functional partners, PfHsp70-z and PfHop, was investigated using a Bio-Rad ProteOn XPR36 surface plasmon resonance (SPR) system (Hercules, CA, USA), as previously described [16,30]. Protein immobilisation was conducted on a GLC chip. As ligands, PfHsp70-1 and its subdomain, PfHsp70-1_NBD_, were immobilised on the chip to achieve 178 and 188 response units, 190 response units for PfHsp70-z, and 197 response units for PfHop, respectively. As analytes, aliquots of PfHsp70-1, PfHsp70-1_NBD_, PfHsp70-z, and PfHop were prepared at 0, 125, 250, 500, 1000 and 2000 nM and injected at 50 μL/min on each horizontal surface. Data collected was double referenced using a buffer blank (buffer without protein) and a channel in which BSA was immobilised as a non-chaperone protein control. The assay was repeated in the presence of nucleotides (either 5 mM ADP or 5 mM ATP) as controls [16,30]. To determine the effects of EGCG on the protein-protein interaction, the assays were repeated in the presence of 2.5 μM EGCG. The SPR data were analysed as previously described [16,30].

### 4.8. Assessment of the Antiplasmodial Effects of EGCG

*P. falciparum* 3D7 parasites were grown and maintained in continuous culture, as previously described [30,34], with minor modifications. The growth of *P. falciparum* 3D7-infected erythrocytes subjected to treatment with varying amounts (0, 0.156, 0.3125, 0.625, 1.25, 2.5, and 5 μM) of EGCG was monitored using the *P. falciparum* lactate dehydrogenase (pLDH) method, as previously described [31]. As a control, Chloroquine, a known antimalarial drug, was used as a positive control in the assay. Serially-diluted EGCG was added to each culture batch. Uninfected erythrocytes that were similarly treated with EGCG were included to cater for possible lysis of erythrocytes exposed to EGCG. A blank consisting of media and EGCG was also set up. *P. falciparum* 3D7-infected erythrocytes that were not exposed to EGCG treatment were used as the viability control, representing ‘100% growth’. Analysis of the growth inhibition assay data was carried out using GraphPad Prism 6 (San Diego, CA, USA). The assay was repeated three times.

## 5. Conclusions

Findings from the current study demonstrate that the green tea constituent, EGCG, directly binds to the ATPase domain of *P. falciparum* Hsp70 resulting in conformational changes that abrogate the chaperone function of the protein. In addition, our study demonstrated that EGCG recognises two distinct Hsp70s (PfHsp70-1 and PfHsp70-z) that are resident in the cytosol of *P. falciparum*. Binding of EGCG to the two proteins also abrogated their interaction with functional associates. We further demonstrated that EGCG exhibits antiplasmodial activity. Our findings suggest that one of the mechanisms by which EGCG manifests antiplasmodial function is through the inhibition of the Hsp70 chaperone function. In addition, EGCG abrogates association of the chaperone with its chaperone/co-chaperone functional network partners. The findings support the utility prospects of EGCG as an antimalarial agent. The distinct affinities exhibited by EGCG for PfHsp70-1 versus PfHsp70-z suggest that EGCG may selectively recognise the two *P. falciparum* cytosol localised Hsp70 molecular chaperones. In the future, it will be important to investigate if EGCG targets parasite Hsp70 without adversely impacting the human Hsp70 pathway.

## Figures and Tables

**Table 1:** Comparative affinities for ATP and EGCG binding to PfHsp70-z and PfHsp70-1.

Protein	ATP *KD* (μM) (±Standard Deviation)	EGCG *KD* (μM) (±Standard Deviation)
PfHsp70-1	3.48 (±0.17)	0.44 (±0.07)
PfHsp70-1_NBD_	3.47 (±0.29)	0.35 (±0.05)
PfHsp70-z	25.3 (±0.33)	2.7 (±0.09)

The standard deviations shown in parenthesis were obtained from at least three independent assays.

**Table 2:** A summary of the kinetics of the ATPase activities of PfHsp70-z and PfHsp70-1 in the presence of EGCG.

**PfHsp70-1**							
EGCG (μM)	0	0.156	0.3125	0.625	1.25	2.5	5.0
*V_max_* (nmol/min/mg)	30.48 (±0.9)	28.22 (±1.5)	29.03 (±0.7)	28.40 (±0.95)	27.47 (±2.01)	29.04 (±0.6)	30.06 (±0.5)
*K_m_* (μM)	384.3 (±0.5)	437.3 (±0.1)	507.7 (±0.7)	655.7 (±0.9)	875.9 (±0.1)	819 (±0.01)	954 (±0.05)
**PfHsp70-z**							
EGCG (μM)	0	0.156	0.3125	0.625	1.25	2.5	5.0
*V_max_* (nmol/min/mg)	20.90 (±0.8)	20.58 (±0.5)	18.46 (±0.3)	19.66 (±0.5)	21.26 (±0.9)	18.88 (±0.6)	17.57 (±0.3)
*K_m_* (μM)	245.4 (±0.5)	316.0 (±0.9)	590.6 (±0.5)	665.6 (±0.5)	970 (±0.9)	1015 (±0.4)	1119 (±0.3)

The standard deviations shown in parenthesis were obtained from at least three independent assays.

**Table 3:** Relative affinities for association of PfHsp70-1 with its interactors in the presence of nucleotide and/or EGCG.

Analyte, Ligand	Nucleotide	*K_D_* (M)	*χ^2^*
PfHsp70-1, PfHsp70-z	ATP	2.41 (±0.2) × 10^8^	1.86
	ADP	1.21 (±0.1) × 10^6^	2.12
	NN	5.98 (±0.5) × 10^5^	3.32
	EGCG	6.10 (±1.8) × 10^4^ *	6.76
PfHsp70-1_NBD_, PfHsp70-z	ATP	2.12 (±0.2) × 10^9^	2.65
	ADP	9.86 (±0.9) × 10^8^	6.52
	NN	1.57 (±0.6) × 10^8^	2.44
	EGCG	1.81 (±0.7) × 10^4^ **	4.03
PfHsp70-1, PfHop	ATP	1.13 (±0.5) × 10^8^	2.15
	ADP	1.72 (±0.5) × 10^9^	2.30
	NN	1.91 (±0.01) × 10^9^	2.17
	EGCG	9.96 (±0.9) × 10^5^ **	6.22
